# Increased Tolerance and Resistance to Virus Infections: A Possible Factor in the Survival of *Varroa destructor*-Resistant Honey Bees (*Apis mellifera*)

**DOI:** 10.1371/journal.pone.0099998

**Published:** 2014-06-13

**Authors:** Barbara Locke, Eva Forsgren, Joachim R. de Miranda

**Affiliations:** Department of Ecology, Swedish University of Agricultural Sciences, Uppsala, Sweden; University of North Carolina, Greensboro, United States of America

## Abstract

The honey bee ectoparasitic mite, *Varroa destructor*, has a world-wide distribution and inflicts more damage than all other known apicultural diseases. However, *Varroa*-induced colony mortality is more accurately a result of secondary virus infections vectored by the mite. This means that honey bee resistance to *Varroa* may include resistance or tolerance to virus infections. The aim of this study was to see if this is the case for a unique population of mite-resistant (MR) European honey bees on the island of Gotland, Sweden. This population has survived uncontrolled mite infestation for over a decade, developing specific mite-related resistance traits to do so. Using RT-qPCR techniques, we monitored late season virus infections, *Varroa* mite infestation and honey bee colony population dynamics in the Gotland MR population and compared this to mite-susceptible (MS) colonies in a close by apiary. From summer to autumn the deformed wing virus (DWV) titres increased similarly between the MR and MS populations, while the black queen cell virus (BQCV) and sacbrood virus (SBV) titres decreased substantially in the MR population compared to the MS population by several orders of magnitude. The MR colonies all survived the following winter with high mite infestation, high DWV infection, small colony size and low proportions of autumn brood, while the MS colonies all perished. Possible explanations for these changes in virus titres and their relevance to *Varroa* resistance and colony winter survival are discussed.

## Introduction

Honey bee (*Apis mellifera*) colonies are declining in the United States and in Europe causing economical stress to apiculture and to the agricultural crop production industries that rely on honey bee pollination [1]. On top of a list of health stressors associated with honey bee colony collapse is the ectoparasitic mite *Varroa destructor* [2], [3]. This parasite has a world-wide distribution and inflicts more damage and higher economic costs than all other known apicultural diseases [4].

The *Varroa* mite causes physical and physiological damage to individual bees while it feeds on bee haemolymph resulting in a reduced live-span [5], reduced learning capability [6] and host immunosuppression [7]. However, *Varroa*-induced colony collapse is more accurately a result of secondary virus infections vectored by the mite that precipitates a progressive epidemic and ultimately colony mortality [8], [9], [10]. Although initially obscure and practically unknown, deformed wing virus (DWV) has become one of the most prevalent viruses world-wide due to its close association with *Varroa* mite infestation [11], [12]. *Varroa*-mediated virus transmission during pupal development is directly responsible for the characteristic wing deformities resulting in flightless adults that die shortly after emerging [12], [13].

In the absence of *Varroa* honey bee viruses can persist as covert infections in the colony maintained through a variety of horizontal and vertical transmission routes between bees [10], [11], [14], [15]. However, with the mite's exponential population growth through the season, increased virus transmission leads to overt infections that in temperate climates usually peak during autumn and winter [9], [16]. The *Varroa*-virus complex will usually result in a virus epidemic causing colony mortality within 2–3 years usually during winter if the mite population is left unmanaged [4]. For this reason the apicultural industry relies heavily on *Varroa* mite population control to prevent virus epidemics from destroying colonies.

However, mite control treatment is not required in Africa and South America to keep honey bee colonies alive. These populations of African and Africanized honey bees are effectively mite-resistant, maintaining lower mite infestation rates than European honey bees in other parts of the world [3]. Furthermore, even though all known bee viruses have been reported in these continents [17], these bees experience no obvious negative health effects [18], [19], [20]. It has been suggested that along with developing mite-resistance these honey bee populations may have also developed a resistance or tolerance to viruses [20].

There are also documented populations of European honey bees, in Europe and North America, that have survived over 10 years with *Varroa* mite infestation but without any kind of mite population control [21], [22], [23]. Studies have revealed that two of these populations in Europe have developed adaptive resistance through natural selection and can limit the mite's population growth within a colony by, through mechanisms not yet fully understood, reducing the mite's reproductive success, *i.e.* the ability for the mite to produce a viable mated female offspring [24]. Reduced mite reproductive success has also been documented for Africanized bees, which are also naturally resistant to *Varroa* mite infestation [3], [25]. However, in none of these populations have the mite-associated virus infection dynamics been examined as a possible explanatory factor for their enhanced survival, which is surprising as it is actually the virus that is the direct cause of mortality in *Varroa*-induced colony collapse.

The aim of this study was to gain better insight into the different factors underpinning survivorship of a honey bee population on the island of Gotland, Sweden that has been surviving uncontrolled mite infestation for over 10 years through natural selection alone [26], [27]. No artificial selection by controlled breeding or beekeeping practices has occurred in this population [27]. Although mite-resistance traits (specifically reduced Varroa mite reproduction) have been identified in this population, the mite infestation rates still border winter mortality thresholds for the region [23], [26] suggesting that there may be other factors contributing to their survival. Specifically we were interested to determine if the mite-resistance adaptations in this population have affected the epidemiological dynamics of mite associated honey bee virus infections, and/or if the Gotland population has developed resistance or tolerance to virus infections during the natural selection process over the last decade, allowing the population to survive winter despite normally lethal *Varroa* infestation rates. This latter possibility is explored here. To accomplish our aim, we screened and monitored late season virus infections, *Varroa* mite infestation and honey bee colony population dynamics in the Gotland mite-resistant (MR) population and compared this to mite-susceptible (MS) colonies that have been regularly managed and previously treated for mite infestation.

## Materials and Methods

### Data and sample collections

Data and samples were collected from 14 mite-resistant (MS) colonies and 11 mite-susceptible (MS) colonies on the island of Gotland, Sweden, (Näsudden: N 57° 07′59,92 E 18° 20′95,38) on July 28 (18°C), August 26 (18°C) and October 7 (12°C), representing the late summer and autumn seasons of 2009. The field site was on private land and permission for the location and activities was obtained from the owner, Åke Lyberg. This study did not involve endangered or protected species. Estimates of the adult bee and brood populations for each colony were made using the Liebefeld estimation method [28]. The *Varroa* mite infestation rates were determined by washing samples of approximately 200 bees with soapy water to dislodge the mites [29]. Bulk samples of 30 adult bees were collected from the brood chamber and stored at −20°C until subsequent virus analysis [30], [31]. Colonies were left in the field over winter and mortality was noted either in the late fall 2009 or in the early spring the next year when colonies were routinely checked. The cause of mortality was in all cases suspected to be *Varroa* mite related due to the status of the colonies in terms of size and visible DWV symptoms from earlier inspections. The dead colonies did not show signs of starvation and did not have symptoms of any other disease.

### Molecular analysis

Total RNA was extracted from the bee samples as described previously [32], using the RNEasy manufacturer's protocol for plant tissues (Qiagen). Eluted RNA was stored as two 25-µl aliquots at −80°C.

To determine which viruses were present in the colonies, a random selection of samples of adult bees from each of the three different time points were screened by reverse transcription-quantitative PCR (RT-qPCR) for 10 honey bee viruses: acute bee paralysis virus (ABPV), black queen cell virus (BQCV), chronic bee paralysis virus (CBPV), deformed wing virus (DWV), Israeli acute paralysis virus (IAPV), Kashmir bee virus (KBV), sacbrood virus (SBV), slow bee paralysis virus (SBPV), Varroa destructor virus 1 (VDV-1), and Varroa destructor macula-like virus (VdMLV). Only the viruses detected in this initial screen (DWV, BQCV, SBV, and KBV) were analysed in the remaining samples. Each sample was also assayed for the mRNA levels of two common internal reference genes, β–actin and RP49 [33], used here to normalize the RT-qPCR data for sample differences in RNA quantity and quality.

The amounts of DWV, SBV, BQCV, KBV, β–actin, and RP49 were determined as previously described [32] using the Bio-Rad iScript One-Step RT-qPCR Kit with SYBR Green detection chemistry, 96-well optical qPCR plates, and the Bio-Rad Chromo4 thermocycler. Three positive controls and one non-template control (nuclease-free H_2_O) were included for each assay. The positive controls were prepared as previously described [32] and covered 6 orders of magnitude difference in concentration. These were used to establish the calibration curves for quantification of the target amounts. The amplification reaction conditions and melting curve analysis for determining the specificity of the amplification products were performed as described previously [32].

### RT-qPCR data conversion, transformation, normalization

The RT-qPCR data were converted to estimated copy numbers of each target RNA per bee as described previously [32], using the calibration curves set up with cloned positive controls for each target. Virus and mite infestation data were log transformed to meet assumptions of normally distributed data for parametric analysis.

### Statistical analysis

All statistical analyses were performed in SAS 9.1 using linear repeated-measures mixed-effects models (SAS proc MIXED) to independently test the effects that mite-resistant colonies compared to mite-susceptible colonies (treatment groups) had on the different viruses, mite infestation, amount of bees in the colony, and the proportion of brood production in the colony. The covariance structure for the repeated factor was selected based on the Aikaike's information criteria [34].

Shapiro-Wilks tests as well as analysis of residuals and equality of variance verified assumptions of normality for the dependent variables [34]. The Satterthwaite method was used to approximate denominator degrees of freedom in all models [34].

## Results

### Bee population parameters

**Figure 1:**
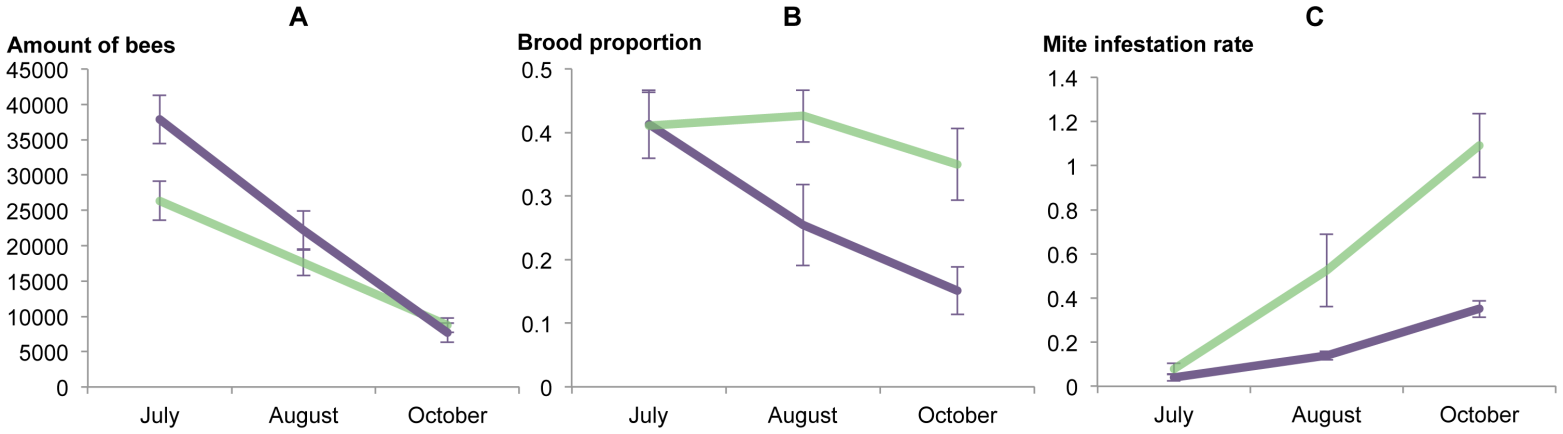
Honey bee colony parameters and mite infestation rates. The progression of the mean values of (A) the total amount of bees and brood in the colony, (B) the proportion of brood production in the colony and (C) the mite infestation rates for the mite-resistant (MR) colonies (purple lines) and mite-susceptible (MS) colonies (green lines) during the late summer to autumn of 2009 on Gotland, Sweden. Standard error bars are shown.

A significant decrease of the colony population size (adult bees and brood) was observed over the sampling dates in all colonies (*F*_2,24.5_ = 12.2, *P* = 0.0002; Fig. 1a). However, although the MR colonies maintained much smaller bee populations than the MS colonies in July rendering an overall significant differences between the two populations (*F*_1,38.2_ = 10.37, *P* = 0.0026), by the end of the season in October the colony sizes were about equal (Fig. 1a). This meant that the rate of decrease was significantly higher for the MS colonies than for the MR colonies. The mite infestation rate was negatively correlated with population size (R = −0.56; Fig. S1a) and had a significant explanatory effect on the total number of bees and brood in the colonies (*F*_1,50.3_ = 5.99, *P* = 0.0179). No correlation or explanatory effect of mites on brood amounts or vice versa was observed.

The proportion of brood in the colony also developed differently for the MR and MS populations during autumn (*F*_1,29.4_ = 5.62, *P* = 0.0245). During July both populations maintained similar proportions of brood but as autumn progressed the MR population reduced its proportion of brood much faster than the MS population (Fig. 1b). SBV titres had a significant explanatory effect on the proportion of brood in the colony (*F*_1,36.7_ = 4.41, *P* = 0.0426) and were positively correlated (R = 0.43; Fig. S1b).

All the MR colonies in this study survived the winter following this study while all the MS colonies perished during the winter. Mortality was determined to be caused by the high mite infestation rates recorded in the fall for all cases.

### Mite infestation

**Figure 2:**
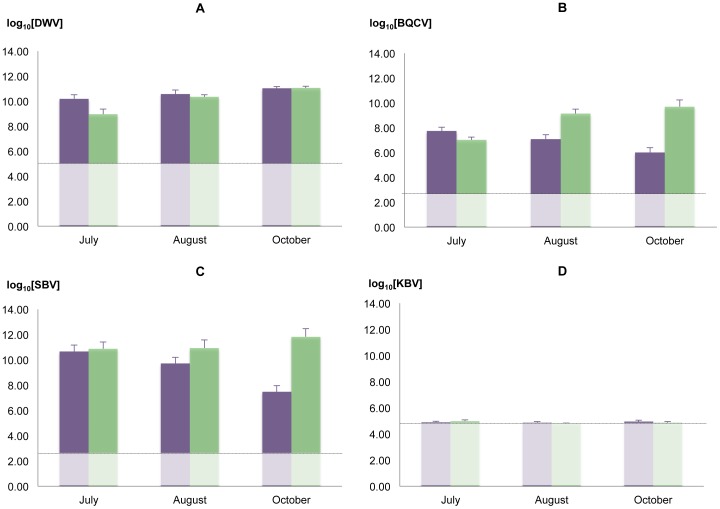
Virus infection dynamics. Virus titres in adult bees (mean values with standard error bars) of (A) deformed wing virus (DWV), (B) black queen cell virus (BQCV), (C) Sacbrood virus (SBV) and (D) Kashmir bee virus (KBV) in mite-resistant (MR) colonies (purple bars) and mite-susceptible (MS) colonies (green bars) from late summer to autumn of 2009 on Gotland, Sweden. The broken line and opaque in each graph area represent the RT-qPCR detection threshold.

The number of mites per adult bee was significantly lower in the MR colonies than in the MS colonies throughout the season (*F*_1,26.9_ = 17.93, *P* = 0.0002). However, this difference developed as summer progresses to autumn since the infestation rates were practically identical between the populations in July (Fig. 1c). For both populations the mite infestation rate increased significantly as the season progressed (*F*_2,38.4_ = 18.58, *P*<0.0001; Fig. 2c).

### DWV

Although across the entire experiment the DWV titres differed significantly between the MR and MS populations (*F*_1,21.9_ = 10.48, *P* = 0.0038), this was almost entirely due to differences between the populations observed in July only, when the MR colonies had slightly higher titres (Fig. 2a). By October the DWV titres were practically indistinguishable between the MR and MS colonies (Fig. 2a). The mite infestation rate had a positive correlation (R = 0.54; Fig. S1c) and significant explanatory effect on the DWV titres (*F*_1,45_ = 8.46, *P* = 0.0056).

### BQCV

Across the entire experiment, the BQCV titres were significantly lower in the MR population than in the MS population in this study (*F*_1,40.7_ = 8.94, *P* = 0.0047; Fig. 2b). However, the more significant finding is the sharply contrasting direction of change in BQCV titre between the two populations. While in July the BQCV titres were very similar for the MR and MS populations, these titres decreased drastically in the MR population as summer progressed to autumn, while in the MS population the BQCV titres increased (Fig. 2b). The different trends were highly significant (*F*_2,41.6_ = 10.64, P = 0.0002). The mite infestation rate did not have any significant explanatory effect on the BQCV titres, nor did either of the colony population parameters; the proportion of brood and the total population size.

### SBV

The MR and MS colonies had significantly different titres of SBV through this study (*F*_1,42.3_ = 6.48, *P* = 0.0146; Fig. 2c) but, as with BQCV, it is the opposite directional trend in the titres for the MR and MS populations as summer progresses to autumn that is the most significant finding (Fig. 2c). Again, these trends are highly significant (*F*_2,36.5_ = 4.33, *P* = 0.0205) and again these are more favorable for the MR population whose SBV titres decrease while those for the MS population increase towards autumn when in summer SBV titres were practically equal between the populations. Also comparable to BQCV, neither the mite infestation rate nor colony population parameters had any significant explanatory effect on the SBV titres.

### KBV

Borderline amounts of KBV were detected in the initial virus screening and so KBV was included in the assays. However, no trends of any kind were noted for this virus in the experimental data as KBV detection stayed around the detection limit (Fig. 2d). Of the 68 experimental samples, 64 did not detect KBV, a detection rate of about 5%, which is within range of the false-positive detection rate for these assays. For this reason, KBV was left out of further analysis.

## Discussion

In temperate climates, the winter season is a major factor in honeybee colony mortality. Colonies prepare themselves for winter by reducing brood production during autumn, resulting in specialized ‘winter bees’ whose primary role is to keep the queen alive in the center of the nest, through heat generation, insulation and to ensure the survival of a sufficient amount of worker bees to raise the first generations of brood the following spring [35]. Winter is a critical period for the colony since in the absence of brood rearing the survival of the colony is entirely dependent on the individual and collective abilities of the overwintering bees to survive extreme conditions for long periods of time. Naturally the health status of the overwintering bees is a critical factor that can mean the difference between colony survival and mortality [36].

Currently the most significant drivers of honeybee colony mortality are the parasitic mite *Varroa destructor* and the virus epidemics it causes [2], [4]. In this study both these factors (*Varroa* infestation and virus infections) were studied simultaneously in both normal ‘mite-susceptible’ (MS) colonies and colonies from a ‘mite-resistant’ (MR) honey bee population that by means of adaptations through natural selection has become able to survive seasonal (winter) mortality despite the presence of uncontrolled mite infestation.

### Population parameters

As has been shown previously, these MR honey bee colonies maintain smaller colonies throughout the summer and autumn seasons, have lower mite infestation rates and cease brood production earlier in autumn than MS honey bee colonies [23], [26], [27]. However by October 2009 in this study, both mite-resistant and mite-susceptible bees had similar colony sizes going into winter. The difference is how they got there and the quality of the winter bees produced, as evidenced by their differing abilities to survive the winter.

A positive correlation between SBV and the proportion of brood in the colony was found in this study and is probably explained simply since SBV is a brood disease and higher brood amounts will mean more virus hosts. Although mite infestation was correlated with the total number of adult bees and brood, no correlation or explanatory effect of mites on brood amount or vice versa was observed. This was probably due to the large differences between the two populations in terms of the mite infestation growth rates and the seasonal trends in brood production.

### Mite infestation

The mite infestation growth rate was slower in the MR colonies than it was in the MS colonies, likely due to the reduced *Varroa* mite reproduction as a mite resistant trait of the bees in this population developed by adaptations that inhibits the mite's population growth [23]. However, the mite infestation rate in the MR colonies in October reached limits above the winter mortality threshold (>0.3 mites/bee) [26], a sufficient mite population to transmit viruses and cause an epidemic [9]. This suggests other factors may be involved in their winter survival such as a tolerance or resistance to virus infection.

A direct epidemiological relationship between *Varroa* mites and BQCV or SBV was not demonstrated in this study. This is consistent with other findings [32], [37], [38], [39]. However, that is not to say that interactions with the mite do not exist; viruses are opportunistic pathogens and as such can also respond indirectly to mite infestation through a general reduction in overall colony and individual bee health.

### DWV

There is no meaningful difference between the MR and MS bee colonies in the DWV titres, either throughout the season or by the time the colonies start their overwintering period. DWV is the principal *Varroa* transmitted virus and usually the immediate cause of *Varroa* mite – associated colony mortality [12]. As expected, our study showed a strong positive correlation between DWV titres and *Varroa* infestation rates, as well as an explanatory effect of mite infestation rates on DWV titres in the statistical analysis. These results complement numerous prior reports on the active relationship between *Varroa* mites and DWV [8], [11], [39], [40], [41]. The significance of high DWV titres to colony winter mortality is well established [36], [42], which was abundantly confirmed by the rapid demise of the MS colonies in our study. Yet the MR colonies survived the winter with equally high DWV titres as the MS colonies. This suggests that resistance to DWV (i.e. reduced DWV titres) was not a factor in the enhanced winter survival of the mite-resistant bees, but that enhanced tolerance to DWV infection (i.e. better survival of DWV infection) may be a factor. There have been similar suggestions that the resistance of Africanized honey bee populations in Latin America to mite infestation could include an increased tolerance to DWV infection, *i.e*. a reduction of the effect of DWV infection rather than a reduction in pathogen load [20]. Elevated tolerance to DWV would relate to other indicators of individual and colony bee health, especially winter bee health, rather than DWV-based indicators. This hypothesis is complicated by, or perhaps partly proven by, the drastic reductions by October in the titres of BQCV and SBV in the mite-resistant bee colonies, compared to the mite-susceptible colonies, from nearly identical infection levels during the height of summer.

### BQCV & SBV

Both BQCV and SBV are virulent diseases of honey bee brood, particularly open brood, and are characterized similarly by larvae that fail to pupate and turn pale yellow to brown with eventually a sac-like appearance [39]. Neither of these viruses causes visible symptoms of infection in adult bees. BQCV is often associated with the gut parasite *Nosema apis* and is occasionally a problem in queen-rearing operations, especially with ageing populations of worker bees used for nursing grafted larvae in queen-less colonies [8], [43], [44], [45]. BQCV is a commonly detected virus, however worker brood rarely become clinically infected with BQCV, as opposed to queen brood, probably because they are not fed as much or for as long time as queen larvae. SBV is also common in colonies but large numbers of diseased larvae are seldom seen.

Individual bees infected with SBV are unable to resist chilling in low temperatures and unable to maintain usual metabolic rates during overwintering [46]. Further, there is a well-documented relationship between SBV infection with reduced longevity of adult bees and foraging and nursing behaviour [46]. SBV infection in adult bees causes a strong aversion to pollen collecting and consumption [8], [46], [47]. This in turn causes accelerated polytheism resulting in bees abandoning (pollen-driven) brood care, premature transition to (nectar) foraging, poor nutritional health in the colony, and reduced individual health and longevity of adult bees. These effects of SBV would critically affect both the adequate rearing of healthy long-lived winter bees, which involves high levels of pollen consumption both as larvae and adults to generate sufficient protein and fat reserves, and would also critically impact pollen collecting in early spring by the winter bees whose task it is to rear the first new brood of the year. Nothing is known of the effect of BQCV infection on adult bees, but if these are similar to those of SBV then their combined effect could be a highly significant factor in the overall capacity of bees to survive winter, with or without mite infestation. Further, their effects could have caused significant pressure leading to selection for adaptive resistance.

### KBV

Of the known honey bee viruses, KBV is one of the most virulent; causing mortality within three days of inoculations in adults or larvae, despite having no specific clinical symptoms [39]. Although its prevalence is generally low, KBV has been detected in neighboring Denmark and throughout Europe [48]. Our data on the detection of KBV is too limited to draw meaningful conclusions. Either the virus was present but was not affected by the experimental treatments, or the data are a statistical consequence of detecting near the detection threshold. We suspect the later since the 5% detection rate is within range of false-positive detection for these assays, at the indicated detection thresholds.

The MR colonies in this study have been exposed to natural selection for over a decade and as a result, in reaction to high mite infestation pressures, colony level adaptations of mite-resistance have developed in these bees by reducing the mite's reproductive success [23]. The present study suggests that their adaptive resistance to *Varroa* mites includes an increased tolerance to DWV infection, through superior overall individual and colony bee health, which may in part be mediated by a functional, seasonal resistance to BQCV and SBV infections resulting in drastically reduced titres of these viruses in adult autumn (overwintering) bees compared to those from MS colonies, with predictable positive benefits both for actual winter survival and early spring pollen foraging and brood rearing. However, the mechanism of this reduction in BQCV and SBV titres is unclear. It is clearly a phenomenon associated with autumn, the cessation of brood production and the production of winter bees, since the virus status of the MR and MS colonies was identical in summer.

Both SBV and BQCV are typically diseases of spring when there is rapid colony expansion and queen rearing, not of autumn. Since BQCV and SBV are primarily brood diseases, the larva of these populations may have become more resistant to these diseases through adaptations, and break the viral cycle at this point resulting in an overall decreased infection level of these viruses.

Another way that the colonies may have adapted to reduce BQCV and SBV infection levels in the colony could be by reducing brood production earlier in autumn, in order to clean out the infection of these brood diseases from the colony prior to winter bee production. Earlier studies on this population have shown that these colonies maintain and survive with lower general colony size, even throughout the summer [23] and this could be an adaptation to keep pathogens at lower levels.

Both the transmission of brood diseases and hygienic behaviour for removing diseased brood are positively affected by the ratio of bees-to-brood. Conversely, hygienic brood behaviour removes infected larvae, *i.e.* sources of infection for transmission, and simultaneously also affects the bee-to-brood ratio. So the earlier decrease in the proportion of brood in the MR colonies could both be a cause and an effect of a differential hygienic behaviour between MR and MS bees that is only expressed during autumn. Effective brood removal has been shown to reduce BQCV and SBV titres within a colony [8]. Even low hygienic behaviour could cause the diseased brood to die in their cells and not be cleaned out of the hive until early spring, thereby limiting the spread of the infection. However, earlier studies have demonstrated that these Gotland mite-resistant colonies are not particularly more or less hygienic than mite-susceptible colonies [23]. Therefore, hygienic brood removal behaviour probably does not play a role in the varying titres of brood viruses that we see in this study between these MR and MS colonies.

In conclusion, the MR population on Gotland, Sweden is able to survive winters with high mite infestation, high DWV infection, small colony size and low proportions of autumn brood compared to MS colonies that all perished over the winter months in the same environmental conditions. The MR colonies also experienced a drastic reduction in SBV and BQCV infections through the autumn while MS colonies experienced an increase of these infections. Specifically how these reductions come about, as well as their significance for the enhanced winter survival of the MR population still has to be demonstrated. Several plausible explanations, based on the current knowledge of the effects of these viruses, have been presented as an initial hypothesis. Although a comprehensive explanation for how this Gotland population of mite-resistant honey bees is able to survive is still lacking, the results of this study, as well as previous work on reduced mite-reproduction in this population [23], suggest that a variety of survival mechanisms may be adopted by these bees. Further work is in progress to investigate if the virus trends observed in this study are consistent over time as well as to explore the wider role that the honeybee microbiome has in the individual and collective longevity of winter bees that enable the mite-resistant colonies on Gotland to survive, when susceptible colonies perish.

## Supporting Information

Figure S1 **Scatter-plots and correlation analysis.** Scatter-plots with correlation trend lines for (A) *Varroa* mite infestation rates vs. the total number of bees and brood in the colony, (B) the amount of brood in the colony vs. the SBV titres and (C) *Varroa* mite infestation vs. the DWV titres.
(TIF)
