# Supplementary figures and images for: Increased Tolerance and Resistance to Virus Infections: A Possible Factor in the Survival of Varroa destructor-Resistant Honey Bees (Apis mellifera)
Source: PLoS One. 2014 Jun 13;9(6):e99998. doi: 10.1371/journal.pone.0099998 (PMC4057421; doi:10.1371/journal.pone.0099998)

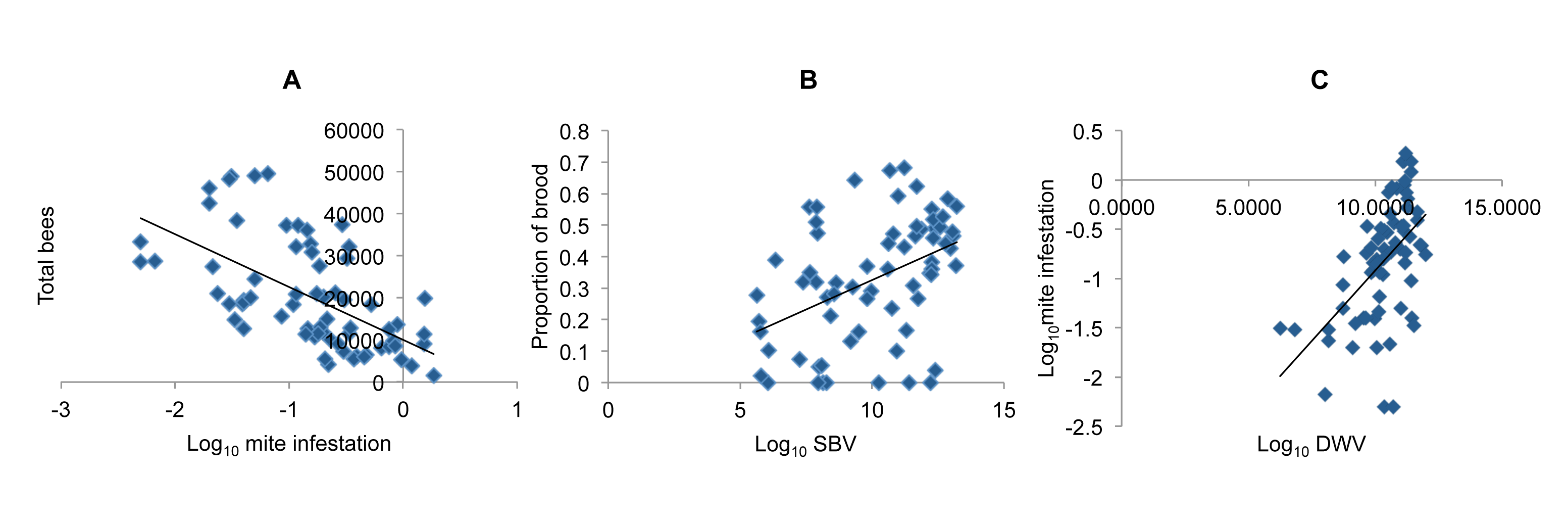

Supplement: Figure S1 — Scatter-plots and correlation analysis. Scatter-plots with correlation trend lines for (A) Varroa mite infestation rates vs. the total number of bees and brood in the colony, (B) the amount of brood in the colony vs. the SBV titres and (C) Varroa mite infestation vs. the DWV titres. (TIF) [file pone.0099998.s001.tif]
